# The era of virtual care: Perspectives of youth on psychiatric virtual appointments in COVID-19 and beyond

**DOI:** 10.1192/j.eurpsy.2022.705

**Published:** 2022-09-01

**Authors:** V. Tsang

**Affiliations:** UBC - Vancouver, BC, Medicine, VANCOUVER, Canada

**Keywords:** adolescent, covid, virtual care, Child Psychiatry

## Abstract

**Introduction:**

In response to COVID-19, paediatric providers have shifted to providing outpatient health care appointments through telehealth.

**Objectives:**

While research has been published previously on this topic, we felt it important to add current Canadian youth perspectives to the mix, specifically on changes due to COVID-19.

**Methods:**

Semi-structured discussions were held on virtual care in June and October 2020 with our youth members, who are patients with various health conditions, aged 13 to 19 years which allowed us to glean their unique opinions regarding virtual care in the midst of a pandemic.

**Results:**

Youth who contributed to this commentary reported that major benefits of virtual care included time savings, ease of access, continuity of care, and ability to participate in health appointments from the comfort of one’s own home without a risk of COVID-19 exposure. These youth also recognized limitations to virtual care, including the inability to complete laboratory or imaging tests, and the lack of physical examination capabilities.

**Conclusions:**

Additionally, they stressed the importance of visual components of virtual appointments and health care providers needing to consider privacy restrictions youth may have. Overall, our cohort of youth feel positive about virtual care and hope care providers can work with youth individually to determine the best solution for them.

**Disclosure:**

No significant relationships.

